# A cone-beam photon-counting CT dataset for spectral image reconstruction and deep learning

**DOI:** 10.1038/s41597-025-06246-4

**Published:** 2025-12-18

**Authors:** Enze Zhou, Wenjian Li, Wenting Xu, Kefei Wan, Yuwei Lu, Shangbin Chen, Gang Zheng, Tianwu Xie, Qian Liu

**Affiliations:** 1 MOE Key Laboratory for Biomedical Photonics, Wuhan National Laboratory for Optoelectronics, Huazhong University of Science and Technology, Wuhan, China; 2 State Key Laboratory of Digital Medical Engineering, School of Biomedical Engineering, Hainan University, Sanya, China; 3 School of Electronic Information and Communications, Huazhong University of Science and Technology, Wuhan, China; 4 Institute of Radiation Medicine, Fudan University, Shanghai, China

**Keywords:** Scientific data, Biomedical engineering

## Abstract

Photon-counting CT has gained significant attention in recent years; however, publicly available datasets for spectral reconstruction and deep learning training remain limited. Consequently, many image process algorithms and deep learning models are developed and validated using simulated rather than real spectral CT data. To address this gap, we present a cone-beam photon-counting CT (PCCT) dataset acquired using a custom-built micro-PCCT system and 15 walnut samples. Each walnut was scanned from four bed positions under dual energy thresholds (15 keV and 30 keV), resulting in a total of 172,800 raw projection images with a resolution of 2063 × 505 pixels. The dataset provides full access to raw multi-energy projections, system parameters, calibration tables, calibration phantom raw projection data and reconstruction code, enabling comprehensive spectral CT studies including spectral CT reconstruction, material decomposition, artifact correction, and deep learning-based methods. It addresses the scarcity of real PCCT datasets for developing and validating data-driven approaches and aims to foster fair and reproducible comparisons across spectral CT image process algorithms.

## Background & Summary

**Fig. 1:**
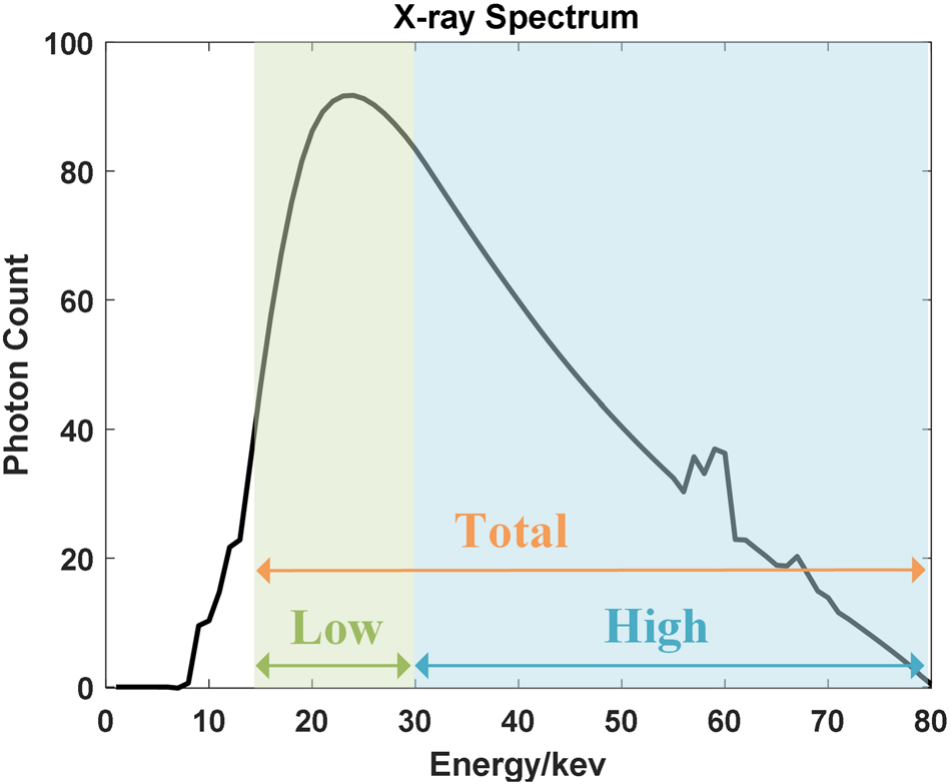
Schematic of the ideal dual-threshold photon-counting detector energy response, with thresholds set at 15 keV and 30 keV, resulting in three energy bins: Total bin ([15 keV, 80 keV]), High bin ([30 keV, 80 keV]), and Low bin ([15 keV, 30 keV]). The X-ray source spectrum (tube voltage: 80 kV) was generated using SpekPy^53^.

With advancements in detector crystal fabrication and integrated circuit technology, photon-counting computed tomography (PCCT), often regarded as the next generation of CT, has garnered increasing attention due to its superior spectral imaging capabilities. Unlike conventional CT that employ energy-integrating detectors (EIDs), PCCT utilizes photon-counting detectors (PCDs), which discriminate and count individual X-ray photons based on their energy levels^1^. By implementing one or more energy thresholds, PCDs can sort incoming photons into different energy bins. As illustrated in Fig. 1, a dual-threshold PCD with energy thresholds of 15 keV and 30 keV can obtain Total bin (15 keV–80 keV) and High bin (30 keV–80 keV) counts in a single measurement, and the Low energy bin (15 keV–30 keV) can then be derived by subtraction. Meanwhile, PCDs offer higher spatial resolution, “zero” electronic noise^2^. These advantages make PCCT highly promising for quantitative material identification^3,4^, enhanced tissue contrast^5–7^ and low-dose imaging^8^.

Deep learning has demonstrated remarkable success in medical image processing, and its applications in PCCT are rapidly expanding. Recent studies have shown its efficacy in noise reduction^9^, artifact correction^10^, image reconstruction^11,12^, and material decomposition^13,14^. However, many deep learning-based spectral CT studies rely on simulated multi-energy data derived from conventional CT images due to the scarcity of real spectral CT datasets. This approach simplifies the complex responses of real systems, such as energy overlap, and noise characteristics, leading to a gap between simulation and reality that limits model generalizability and applicability in real PCCT systems. In addition, many artifact correction methods^15,16^ and advanced spectral reconstruction algorithms^17,18^ require access to raw projection data and precise system geometry, which are often not readily available to researchers. At the same time, instead of relying solely on image-domain denoising, recent studies have begun to make use of projection data as well. For instance, Chao *et al*. demonstrated that incorporating projection data into dual-domain models can further enhance the performance of deep learning-based CT denoising^19^. Access to spectral projection data is therefore essential not only for developing advanced reconstruction algorithms, but also for enabling the development of end-to-end deep learning frameworks in spectral CT, which is key to achieving further improvements in PCCT image quality.

Several CT datasets with projection data are publicly available, such as the cone-beam walnut dataset^20^, the LoDoPaB-CT dataset^21^, and the 2DeteCT dataset^22^, among others^23–25^. Additionally, challenge datasets like the 2016 Mayo Clinic Low Dose CT Challenge^26^ and its extensions^27^ have been widely used. However, these datasets are limited to conventional CT and are not suitable for spectral CT algorithm development. Publicly available spectral CT datasets remain scarce. To our knowledge, the only notable example is the “2022 AAPM Grand Challenge on Deep-Learning Spectral CT Image Reconstruction,” which provides 1,000 simulated 2D breast CT data and tissue-map pairs^28^; However, the dataset is based on simulated data rather than actual spectral CT scans. Other studies have released limited photon-counting CT (PCCT) datasets^29–32^, but these are often task-specific, small in scale, and lack raw projection data. The Multi-Channel CT Reconstruction (MCR) Toolkit^33^ also includes a few spectral CT datasets, but their limited size makes them inadequate for deep learning model training and validation. Therefore, there is currently a lack of a real PCCT dataset that includes raw projection data and is suitable for training deep learning models.

To fill this gap, we constructed and released a walnut photon-counting CT dataset acquired using a custom cone-beam micro-PCCT system. The dataset contains scans of 15 walnuts (this choice will be discussed in the next section), each imaged from four different bed positions using circular trajectories to ensure full field-of-view (FOV) coverage and reduce cone-beam artifacts. In total, the dataset comprises 172,800 photon-counting projection images (15 walnuts × 4 positions × 1440 views × 2 energy thresholds) with a resolution of 2063 × 505 pixels. Alongside the projection data, we provide all necessary acquisition geometry parameters, calibration tables, and reconstruction code, enabling users to perform various spectral CT image reconstruction tasks, including material decomposition, and virtual monoenergetic imaging. This dataset supports a wide range of research topics, including detector defect correction, ring artifact removal, denoising, sparse-view spectral reconstruction, and material decomposition. It offers researchers a real, multi-domain spectral CT platform that facilitates fair comparison across algorithms and practical application of deep learning techniques in photon-counting CT.

## Methods

### Samples

**Fig. 2:**
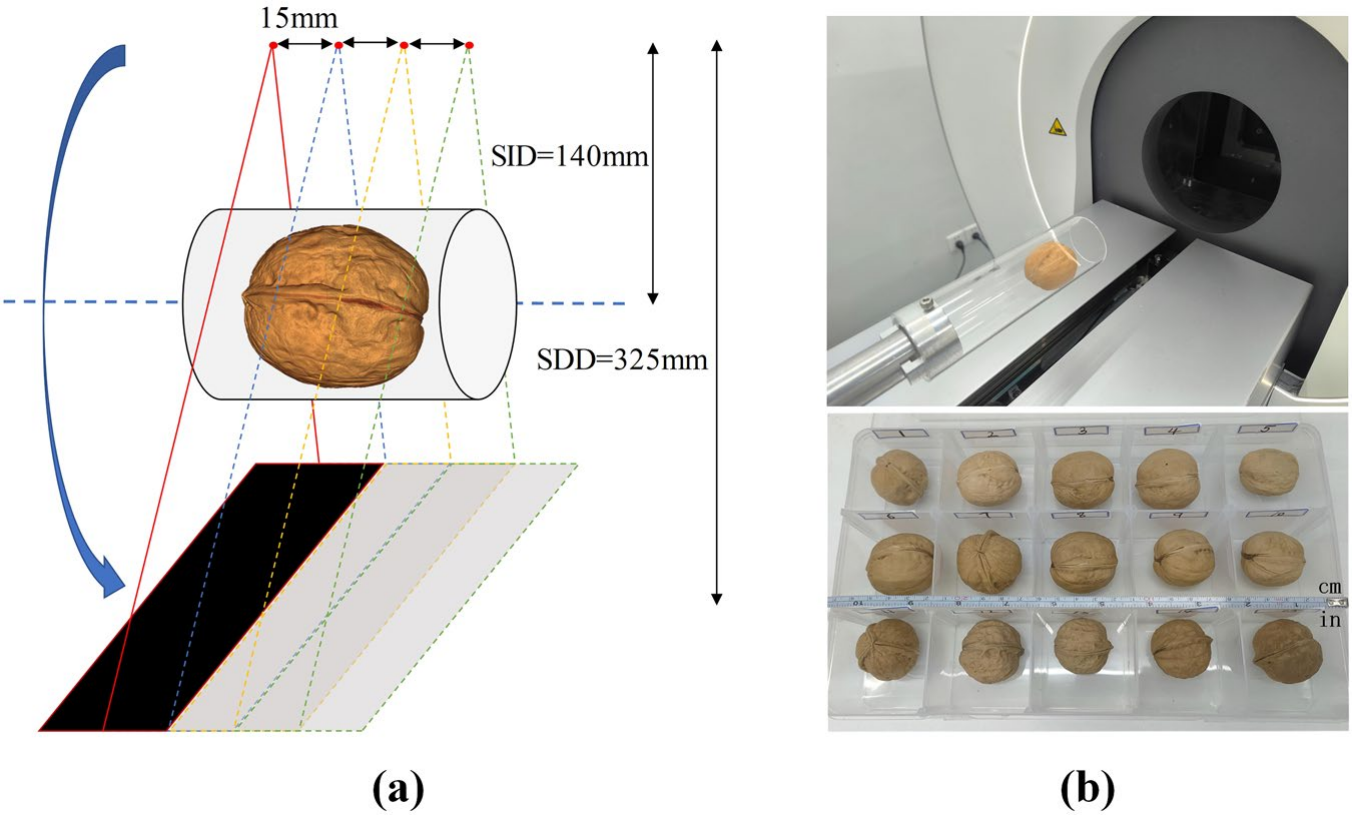
Scanning geometry for walnut imaging. (**a**) Schematic of the scanning trajectories: four circular orbits were used to ensure complete coverage of each walnut. (**b**) The actual Micro-PCCT system used for walnut imaging (top), and 15 walnut samples (bottom).

Photon-counting CT enables multi-energy imaging and thus supports spectral imaging applications such as material decomposition. To fully demonstrate the advantages of spectral imaging, it is important to select samples with rich and heterogeneous material composition. Walnuts have been widely used as imaging subjects in CT research^20,25^ due to their unique anatomical structure, which includes a hard, symmetric shell, soft and irregular inner tissue, and internal air cavities. These multi-scale features resemble those found in animal head models, making walnuts well-suited for spectral PCCT imaging. Deep learning models rely on large and diverse datasets to effectively learn high-level image features; small or homogeneous datasets often lead to overfitting. To address this, we collected a dataset comprising 15 walnuts with variations in cultivar, shape, and size (diameters ranging from 3 cm to 4.5 cm). Walnuts 1–10 were obtained from Aksu, Xinjiang, China, and are characterized by thinner and smoother shells with a more rounded appearance, while walnuts 11–15 collected from the Xinjiang Uygur Autonomous Region, China, have thicker and rougher shells, as shown in Fig. 2(b). The internal kernel structures of the walnuts also vary considerably, further ensuring the overall diversity of the dataset. Due to the limited detector width of our PCCT system, each walnut was scanned at four different bed positions to ensure complete coverage and minimize edge-related cone-beam artifacts, as illustrated in Fig. 2(a).

### Micro-PCCT and acquisition parameters

The micro photon-counting CT (Micro-PCCT) system used in this study was jointly developed by Hainan University and United Imaging Life Science Instrument (LSI, Wuhan, China). It employs a translate-rotate scanning geometry, in which the gantry rotates around a stationary object to reduce motion-induced artifacts during imaging (Fig. 2(b)). The X-ray source (L10321, Hamamats, Japan) operates with a maximum tube voltage of 100 kV and a maximum power of 20 W, featuring a minimum focal spot size of 5 µm. The photon-counting detector (THOR-FX20, XCounter, Sweden) has an original resolution of 2063 × 513 pixels with a pixel size of 100 × 100 µm, and supports two independently adjustable energy thresholds. To avoid marginal artifacts, four detector rows were cropped from both the top and bottom edges, resulting in an effective resolution of 2063 × 505 pixels. Each energy channel uses a 12-bit counter capable of recording up to 4096 photons per acquisition.

Due to the limited detector width, each walnut was scanned at four axial bed positions along circular trajectories, spaced 15 mm apart, to ensure full coverage (Fig. 2(a)). The field of view (FOV) was set to 80 mm to encompass the entire walnut. Scans were performed in continuous mode, acquiring 1440 projections per circular trajectory with an angular increment of 0.25°. The source-to-isocenter distance (SID) and source-to-detector distance (SDD) were 140 mm and 325 mm, respectively. To maximize photon flux, the X-ray source was operated at 80 kV and 200 µA, and a 0.5 mm aluminum filter was used to suppress low-energy photons. The detector thresholds were set to 15 keV and 30 keV. The exposure time per projection was 70 ms to keep photon counts within the detector’s dynamic range. Each scan generated two photon-counting projections: a total energy bin (15–80 keV) and a high-energy bin (30–80 keV). The low-energy bin (15–30 keV) was derived by subtracting the high-energy bin from the total. All walnuts were scanned using the same acquisition protocol, as summarized in Table 1. Flat-field (air) projections were acquired prior to sample scanning for air normalization correction during image reconstruction.

**Table 1:** Summary of the acquisition parameters used.

Para	Value
Tube voltage	80 kV
Tube current	200uA
Filter	0.5 mm AL
Detector rows	2063
Detector columns	505
Detector pixel size	100μm
Energy threshold 1	15kev
Energy threshold 2	30kev
Exposure time	70 ms
Field of view	80 mm
Scan mode	Continuous
Number of projections per circle	1440
Source to object distance	140 mm
Source to detector distance	325 mm
Number of Walnuts	15

### Correction and reconstruction

**Fig. 3:**
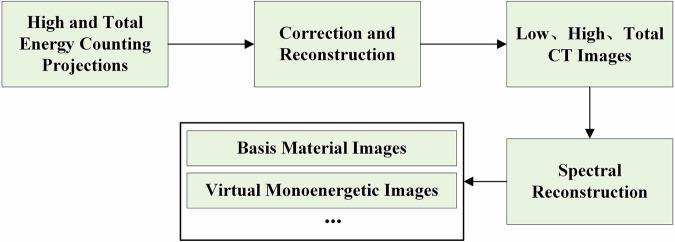
The workflow for reconstructing CT and spectral images using the proposed dataset.

This dataset provides raw photon-counting projection images acquired at high and total energy thresholds. To obtain reconstructed CT slices and spectral images, further correction and reconstruction steps are required. Fig. 3 illustrates the overall reconstruction workflow.

#### Energy bin subtraction and air correction

The photon-counting detector used in this study supports two energy thresholds, enabling the acquisition of photon counts in two energy bins per scan: Total (15–80 keV) and High (30–80 keV). The Low energy bin (15–30 keV) was derived by subtracting the High bin from the Total bin, i.e., Low = Total − High.

After obtaining the photon-counting projections in all three energy bins, air correction was applied to compute the logarithmic projection values for each energy level, using the following equation:

1 $${{\rm{P}}}_{{\rm{E}}}=-{\rm{In}}\left(\frac{{{\rm{I}}}_{{\rm{E}},{\rm{obj}}}}{{{\rm{I}}}_{{\rm{E}},{\rm{air}}}}\right)$$

where ${{\rm{I}}}_{{\rm{E}},{\rm{obj}}}$ and ${{\rm{I}}}_{{\rm{E}},{\rm{air}}}$ are the photon-counting projections of the object and air, respectively, at energy bin E, and ${{\rm{P}}}_{{\rm{E}}}$ is the air-corrected log projection.

#### Photon counting detector non-uniformity correction

**Fig. 4:**
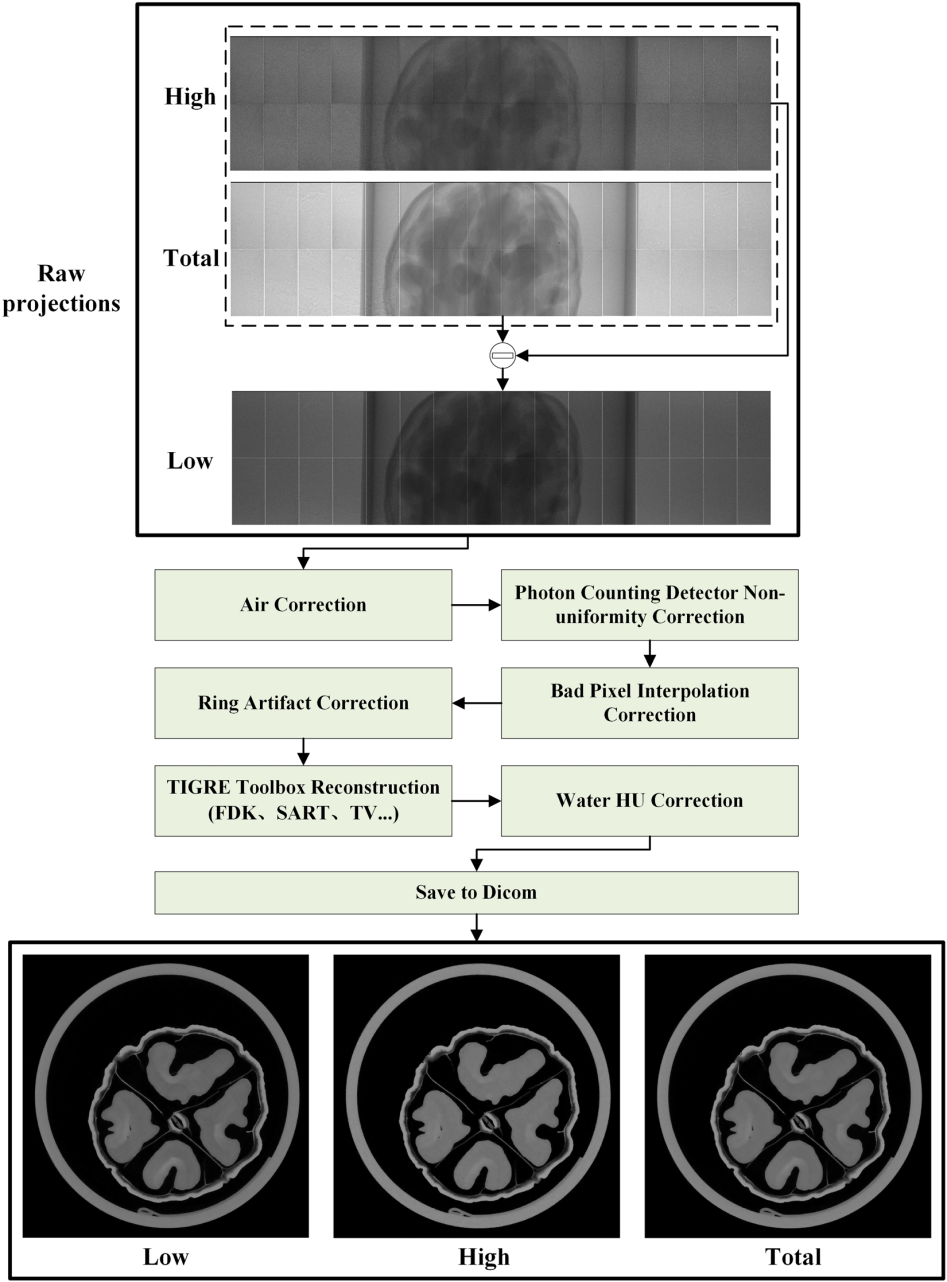
The workflow of photon-counting CT reconstruction. The projection images are displayed with a window of [1, 4000] photons, and the reconstructed CT images are shown with a window of [−1000, 1000] HU.

**Fig. 5:**
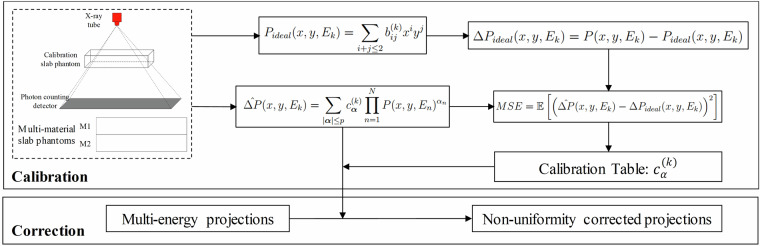
Workflow of detector non-uniformity correction.

As shown in the “Raw Projections” of Fig. 4, the acquired projection images in each energy bin exhibit noticeable non-uniformity artifacts, primarily caused by variations in detector response between tiles and between individual pixels. Without correction, such non-uniformities result in severe ring artifacts in the reconstructed CT images. To address this issue, we applied our proposed Signal-to-Uniformity Error Polynomial Calibration (STEPC)^34^ algorithm to correct for detector non-uniformity in photon-counting CT. As illustrated in Fig. 5, the method consists of two main stages: calibration and correction.

**Fig. 6:**
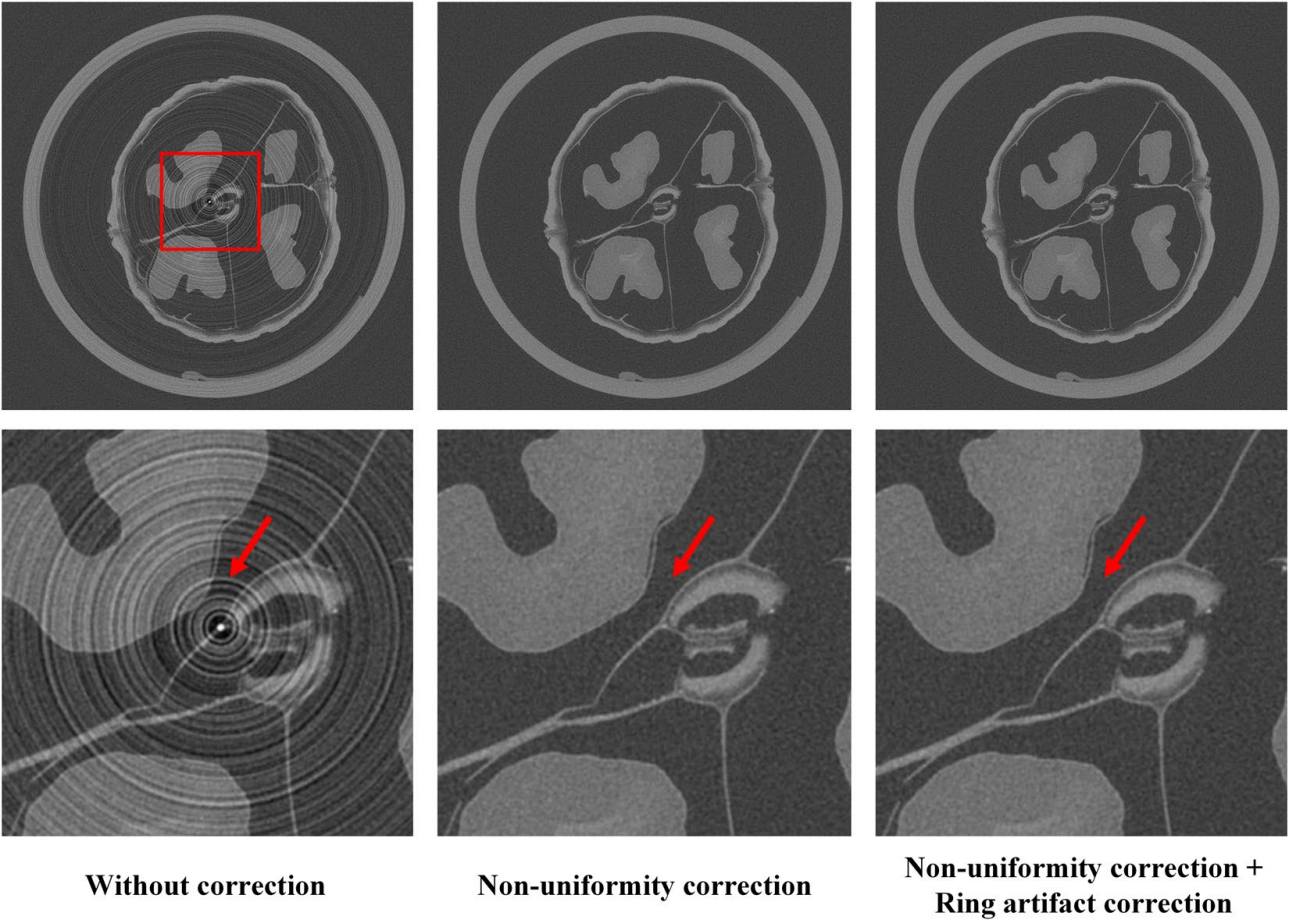
Performance of non-uniformity and ring artifact correction. Left: without correction; Middle: with non-uniformity correction; Right: with both non-uniformity and ring artifact correction. Display window: [−2000, 2000] HU.

In the calibration stage, homogeneous slab phantoms, including PMMA slabs of varying thicknesses (5, 10, 15, 20, 30, and 40 mm) and aluminum slabs (0.5, 1, 1.5, 2, 3, 4, and 5 mm), are combined and scanned to generate multi-energy flat-field projection data. For each energy bin, a 2D second-order polynomial surface is fitted to model the ideal detector response. The residuals between the measured and fitted values are computed as the ideal uniformity error. A nonlinear multi-energy polynomial model is then used to predict this non-uniformity error. The model coefficients are determined by minimizing the mean squared error (MSE) between the predicted and ideal uniformity errors. In the correction stage, during real object PCCT scans, the trained multi-energy residual model is used to predict and correct the non-uniformity in each projection. This process significantly improves projection uniformity and reduces ring artifacts in the reconstructed images. As illustrated in Fig. 6, the non-uniformity corrected result shows a significant reduction in ring artifacts compared to the uncorrected data, demonstrating the effectiveness of the correction processing.

#### Bad pixel interpolation correction

Furthermore, we perform bad pixel correction using a provided bad pixel index table, which includes pre-identified abnormal response pixels as well as gap pixels between detector tiles. In addition, for each sample’s raw projections, pixels with photon counts below 5 or above 4090 are identified as new bad pixels. These bad pixels are then corrected using one-dimensional linear interpolation.

#### Ring artifact correction

**Fig. 7:**
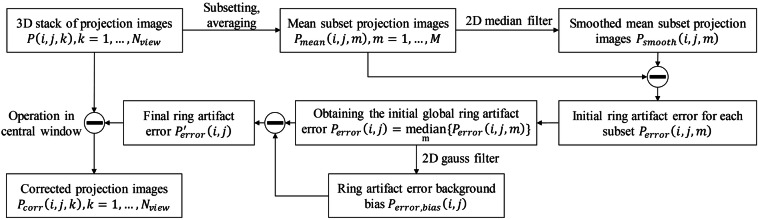
Workflow of ring artifact correction.

Due to the significant non-uniformity response of current photon-counting detectors, prior non-uniformity correction may still be insufficient to fully eliminate artifacts. To address this, we improved upon the ring artifact correction methods proposed by Ketcham *et al*.^35^ and Eldib *et al*.^36^, and developed a projection-domain ring artifact correction method, whose workflow is illustrated in Fig. 7 and consists of the following steps:

**Step 1:** Divide all projection images $P\left(i,j,k\right)$ across angles into *M* subsets, i and j are pixel coordinate indices, and k is the angular index.

**Step 2:** For each subset, compute the mean projection ${P}_{{mean}}\left(i,j,m\right)$, m is the subset index, then apply a 2D median filter to obtain a smoothed mean projection ${P}_{{smooth}}\left(i,j,m\right)$.

**Step 3:** Calculate the initial ring artifact error for each subset by subtracting the smoothed projection from the mean:

2 $${P}_{{error}}(i,j,m)={P}_{{mean}}(i,j,m)-{P}_{{smooth}}(i,j,m).$$

**Step 4:** Compute the median of all subsets errors to obtain the initial grobal ring artifact error:

3 $${P}_{{error}}\left(i,j\right)=\mathop{{median}}\limits_{m}{P}_{{error}}\left(i,j,m\right).$$

**Step 5:** Smooth ${P}_{{error}}\left(i,j\right)$ using a 2D Gaussian filter to get the ring artifact error background bias ${P}_{{error},{bias}}(i,j)$, then calculate the final ring artifact error as:

4 $${P}_{{error}}^{{\prime} }(i,j)={P}_{{error}}(i,j)-{P}_{{error},{bias}}(i,j).$$

**Step 6:** Finally, all projection images is corrected by subtracting the final ring artifact error:

5 $${P}_{{corr}}\left(i,j,k\right)=P\left(i,j,k\right)-{P}_{{error}}^{{\prime} }\left(i,j\right).$$

Dividing all projections into subsets can reduces the introduction of new artifacts caused by object edges^35^. Additionally, since ring artifacts are typically more severe near the center of the image, the correction can be selectively applied to the central-channel window. As illustrated in Fig. 6, the proposed ring artifact correction further suppresses ring artifacts in the reconstructed images.

#### TIGRE Toolbox Reconstruction

After preprocessing, the projection data were reconstructed using the open-source TIGRE toolbox^37,38^, a powerful and feature-rich platform for CT reconstruction. TIGRE supports both MATLAB and Python environments and offers a variety of analytical and iterative reconstruction algorithms, including FDK, SART, CGLS, and MLEM, as well as 3D total variation (3D-TV) denoising. All algorithms are CUDA-accelerated and can be executed on single or multiple GPUs. The toolbox offers flexible reconstruction options. For example, users can selectively reconstruct sparse-angle projection subsets to simulate low-dose conditions for deep learning-based sparse-view reconstruction tasks. Alternatively, high-quality images obtained via iterative reconstruction can serve as ground truth for supervised learning. Each circular scan trajectory of every walnut is accompanied by complete acquisition parameters, including actual source-to-isocenter distance (SID), source-to-detector distance (SDD), and individual projection angles.

Users may freely customize the reconstruction field of view (FOV) and resolution. As an example, an FDK reconstruction of size 1000 × 1000 × 300 voxels covering a 50 × 50 × 15 mm³ FOV with all 1440 projection angles (full dose) takes approximately 90 seconds on an NVIDIA RTX 2060 SUPER GPU. Since material decomposition is highly sensitive to image noise, we apply 3D-TV denoising to the FDK-reconstructed volumes; 100 iterations require only about 8 seconds. Further details on the reconstruction pipeline and available code can be found in the “Code Availability” section.

#### HU correction and dicom saving

The reconstructed CT volumes represent linear attenuation coefficients ${\mu }_{{obj}}$, which are not ideal for analysis and storage. To address this, we converted the data to Hounsfield Units (HU) and saved them in DICOM format. The conversion was performed using the standard HU formula:

6 $${{\rm{CT}}}_{{obj}}=\frac{{\mu }_{{obj}}}{{\mu }_{{water}}}\ast 1000-1000$$

To enable accurate HU calibration, we pre-scanned a water cylinder phantom and calculated the HU scaling factor $\frac{1000}{{\mu }_{{water}}}$ for each energy window. These scaling factors were compiled into a calibration table and used for HU correction. The HU corrected data were then exported as DICOM files for standardized visualization and analysis.

#### Spectral reconstruction

Photon-counting CT enables the acquisition of multi-energy images, which in turn supports advanced spectral imaging applications such as material decomposition and virtual monoenergetic imaging. Material decomposition assumes that each voxel consists of a linear combination of two basis materials. Under this model, the linear attenuation coefficients at different energies can be expressed as:

7 $$\left[\begin{array}{c}{{\rm{\mu }}}_{L}\\ {\mu }_{H}\end{array}\right]=\left[\begin{array}{cc}{\mu }_{L,1}^{m} & {\mu }_{L,2}^{m}\\ {\mu }_{H,1}^{m} & {\mu }_{H,2}^{m}\end{array}\right]\left[\begin{array}{c}{\rho }_{1}\\ {\rho }_{2}\end{array}\right]$$

Here ${{\rm{\mu }}}_{L}$ and ${\mu }_{H}$ are the measured linear attenuation coefficients at low and high energy thresholds, respectively; ${\mu }_{L/H,1/2}^{m}$ are the mass attenuation coefficients of the two basis materials at the corresponding energy levels; ${\rho }_{1}$ and ${\rho }_{2}$ are their densities to be estimated. The low and high energy images are chosen due to their minimal spectral overlap, which improves decomposition robustness. To simplify the decomposition in the image domain, we normalize the equations by the linear attenuation coefficients of water ${\mu }_{L/H,{water}}^{m}$, converting them into Hounsfield Units (HU). This yield:

8 $$\left[\begin{array}{c}\frac{{{\rm{\mu }}}_{L}}{{\mu }_{L,{water}}^{m}}\times 1000\\ \frac{{\mu }_{H}}{{\mu }_{H,{water}}^{m}}\times 1000\end{array}\right]=\left[\begin{array}{cc}\frac{{\mu }_{L,1}^{m}}{{\mu }_{L,{water}}^{m}}\times 1000 & \frac{{\mu }_{L,2}^{m}}{{\mu }_{L,{water}}^{m}}\times 1000\\ \frac{{\mu }_{H,1}^{m}}{{\mu }_{H,{water}}^{m}}\times 1000 & \frac{{\mu }_{H,2}^{m}}{{\mu }_{H,{water}}^{m}}\times 1000\end{array}\right]\left[\begin{array}{c}{\rho }_{1}\\ {\rho }_{2}\end{array}\right]$$

**Fig. 8:**
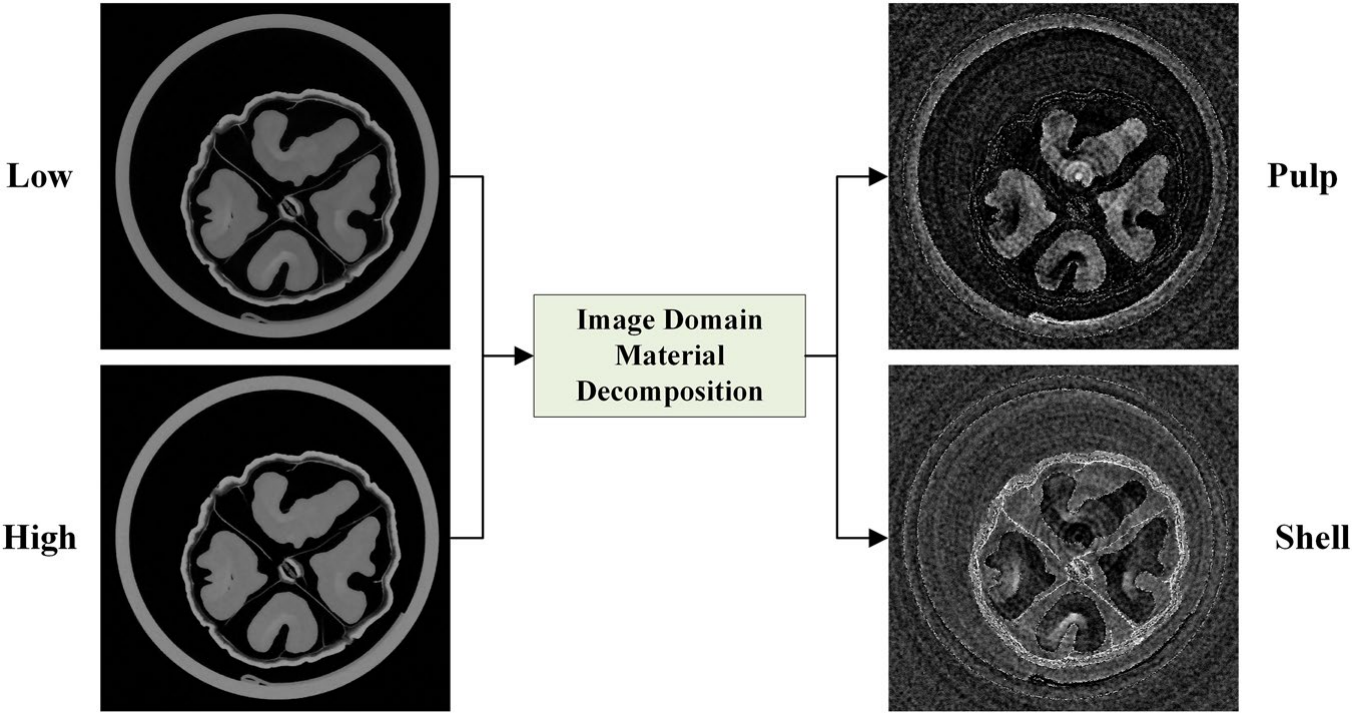
Material decomposition of walnut shell and pulp in the image domain. CT images display window: [−1000, 1000] HU, material decomposition maps display window:[−1000, 5000] mg/cm^3^.

To better demonstrate the material decomposition capability in walnuts, we selected walnut shell and walnut pulp as the two basis materials. Their densities and mass attenuation coefficients were experimentally calibrated in advance, yielding HU-normalized decomposition matrices:$\,\frac{{\mu }_{L/H,{pulp}/{shell}}^{m}}{{\mu }_{L/H,{water}}^{m}}\times 1000$. These calibration matrices were then used for image-domain decomposition via the following equation. The resulting material-specific maps are shown in Fig. 8.

9 $$\left[\begin{array}{c}{\rho }_{1}\\ {\rho }_{2}\end{array}\right]={\left[\begin{array}{cc}\frac{{\mu }_{L,1}^{m}}{{\mu }_{L,{water}}^{m}}\times 1000 & \frac{{\mu }_{L,2}^{m}}{{\mu }_{L,{water}}^{m}}\times 1000\\ \frac{{\mu }_{H,1}^{m}}{{\mu }_{H,{water}}^{m}}\times 1000 & \frac{{\mu }_{H,2}^{m}}{{\mu }_{H,{water}}^{m}}\times 1000\end{array}\right]}^{-1}\left[\begin{array}{c}\frac{{{\rm{\mu }}}_{L}}{{\mu }_{L,{water}}^{m}}\times 1000\\ \frac{{\mu }_{H}}{{\mu }_{H,{water}}^{m}}\times 1000\end{array}\right]$$

**Fig. 9:**
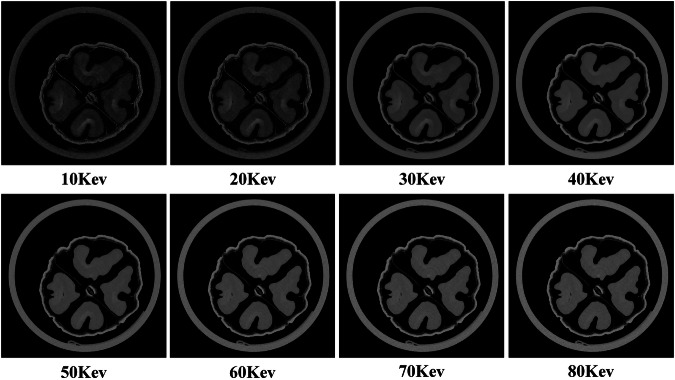
Virtual monoenergetic images (VMIs) of the walnut at different energy levels, display window: [−1000, 1000] HU.

Virtual monoenergetic images (VMIs) of the walnuts can also be generated at different energy levels, as illustrated in Fig. 9. This process begins with material decomposition into water and hydroxyapatite (HAP). The material decomposition coefficients for water and HAP were obtained from calibration data acquired with the water cylinder phantom and the QRM HAP rod insert phantom. The linear attenuation coefficient at an arbitrary energy E is then synthesized using the known mass attenuation coefficients of water and HAP from the NIST database^39^, according to the following equation:

10 $${\mu }_{E}={\mu }_{E,{water}}^{m}{\rho }_{{water}}+{\mu }_{E,{HAP}}^{m}{\rho }_{{HAP}}$$

The resulting attenuation maps are subsequently converted to Hounsfield Units (HU). VMIs allow observation of energy-dependent attenuation differences across materials, providing enhanced tissue contrast and richer spectral information.

## Data Records

The dataset is available at Zenodo^40–47^, consists of four main components: (1) CalibrationTable.zip (~62.5 MB), (2) CalibrationPhantomData.zip (~9.2GB), (3) Reconstructions.zip (~19.0 GB), and (4) complete projection data for individual walnuts, shared as 15 separate ZIP files (Walnut_1.zip - Walnut_15.zip; ~17.9 GB per file). The CalibrationTable.zip includes all necessary calibration datas required for preprocessing, reconstruction, and material decomposition. The CalibrationPhantomData.zip contains projection data acquired from calibration phantoms, including PMMA and aluminum slabs, a water cylinder, and QRM HAP rods. The Reconstructions.zip provides exemplary reconstructed spectral images for Walnut 1. The ZIP file for the sample k, Walnut_ < k > .zip file contains the full set of raw projection images along with the corresponding acquisition parameters. Due to the dataset’s total size (~296.8 GB), files were uploaded to Zenodo in partitioned bundles, each assigned a unique DOI: calibration table&sample1^40^, sample2-3^41^, sample4-5^42^, sample6-7^43^, sample8-9^44^, sample10-11^45^, sample12-13^46^, sample14-15^47^. All files are accessible via Zenodo’s web interface and can be downloaded individually.

The CalibrationTable.zip file contains the following 13 components:

- **air_table_low.raw, air_table_high.raw, air_table_total.raw:** Multi-energy flat-field (air) projection data required for air correction during reconstruction, saved in 16-bit unsigned raw format with dimensions Width = 2063, Height = 505. The air projection is the average of 1440 views to reduce the impact of noise.
- **badchannelIndexAll.data:** Index list marking all bad pixels, stored as float32.
- **STEPC_table_low.data, STEPC_table_high.data, STEPC_table_total.data:** STEPC non-uniformity correction polynomial tables for all energy bins, stored as float32 with dimensions 2063 × 505 × 5.
- **HU_water_table.mat:** Calibration table required for water HU correction.
- **WalnutMDTable.mat:** Calibration table required for walnut pulp–shell material decomposition.
- **Watercali.mat, HAPcali.mat:** Calibration tables for water–HAP material decomposition.
- **H2O_massAttenuationCoeff.mat, HAP_massAttenuationCoeff.mat:** Mass attenuation coefficients of water and HAP, used to virtual monoenergetic imaging.

Each Walnut_ < k > .zip file contains the raw projection data for both the high and total energy thresholds, along with system acquisition parameters for each circular scan. Each walnut was scanned at four bed positions, with corresponding subfolders: “Walnut_ < k > \couch_ < i > ”, Each “couch_ < i > ” folder contains two subfolders: “High” and “Total”, representing two energy thresholds. Each of these includes:

- **AcqPara.mat:** Acquisition parameters including tube voltage, tube current, SID, SDD, projection angles, and all information required for reconstruction.
- **proj_ < j > .raw:** projections at angle *j* (j = 00001–01440), saved in 16-bit unsigned raw format with dimensions Width = 2063, Height = 505.

The CalibrationPhantomData.zip file contains phantom projection data acquired for generating calibration tables. It includes three main folders: “PMMA_AL_slabs”, “Water_Phantom”, and “HAP_Phantom”. Their detailed composition and descriptions are as follows:

- **PMMA_AL_slabs folder:** Used for generating the STEPC correction tables, this folder contains an “AcqPara.mat” file storing imaging acquisition parameters and 56 subfolders named “PMMA_ < m > _AL_ < n > ”, where m and n indicate the thicknesses of PMMA and aluminum (in mm), representing different PMMA and AL slab combinations. Each subfolder includes two files: “proj_high.raw” and “proj_total.raw”, corresponding to projections acquired at the high and total energy thresholds, respectively. Each projection is the average of 600 acquired frames to reduce the impact of noise.
- **Water_Phantom folder:** Contains the raw projections of the water cylinder phantom, used for generating the water HU calibration table. It includes two subfolders: “High” and “Total”. Each subfolder contains an “AcqPara.mat” file and projection files “proj_ < j > .raw”, where j represents the projection angle index (j = 00001–01440).
- **HAP_Phantom folder:** Contains the raw projection data of the QRM HAP rod insert phantom (comprising 50, 100, and 200 mg/cm³ HAP rods embedded in a PMMA cylindrical holder), used for generating the HAP calibration table. It also has two subfolders, “High” and “Total”, each containing an AcqPara.mat file and projection files “proj_ < j > .raw” (j = 00001–01440).

The Reconstructions.zip file contains example reconstructed spectral images. we provide full-angle (label “Dose_1”) FDK reconstructions (with Hann filter), followed by 3D total variation (TV) denoising (TV_niter = 100, TV_lambda = 20) for Walnut 1 in “Reconstructions\Walnut_1\FDK_Dose_1_hann_TV_100_20”, This sub-folder includes:

- **Low, High and Total subfolders:** Reconstructed volumes for each energy bin.
- **MD_Pulp_Shell\Pulp and MD_Pulp_Shell\Shell subfolders:** Material decomposition images of the walnut pulp and shell.
- **VirtualMonoImg\ < e > Kev subfolders:** Virtual monoenergetic images at energy level e **kev**.

Each circular orbit is reconstructed into a volume of size 1000 × 1000 × 300, producing 1200 DICOM slices per walnut (4 beds × 300 slices). Each subfolder contains 1200 DICOM images named: <n>.dcm (n = 00001–01200). It should be noted that all raw projection data are uncorrected, with only four detector rows cropped from the top and bottom edges. All the “.mat” files can be directly loaded in MATLAB using the “load” function.

## Technical Validation

The Micro-PCCT system used in this study requires regular maintenance and calibration. Prior to each walnut scan, the container position is pre-defined and physically marked. Each walnut is carefully aligned with the center of this marker to ensure it remains within the system’s field of view (FOV) throughout the scaning. To ensure high reconstruction resolution, geometric calibration is performed before data collection to determine accurate system geometry parameters. For non-uniformity calibration, projection data are pre-acquired using combinations of PMMA and aluminum slabs with varying thicknesses. These calibration data are used to compute pixel-wise residual error model parameters for non-uniformity correction. Detailed procedures are described in the STEPC method paper^34^.

Bad pixel correction requires prior identification of defective pixel indices, including both the physical gaps between detector tiles, and the pixels with abnormal responses. In this dataset, any pixel with photon counts less than 5 or greater than 4090 is also considered faulty and is corrected using one-dimensional linear interpolation.

The file “**WalnutMDTable.mat**” contains calibration factors for walnut pulp and shell material decomposition, derived from the average CT values of the respective components. Users may also generate their own table by segmenting and analyzing the reconstructed CT images. The file “**Watercali.mat”** is generated by scanning a cylindrical water phantom and computing the mean CT value within the water region. The “**HAPcali.mat**” file is obtained by scanning the QRM hydroxyapatite (HAP) calibration rods with varying densities (50, 100, 200 mg/cm^3^), enabling accurate calibration of HA attenuation across different energy bins. The energy-dependent mass attenuation coefficients for both water and HAP used in virtual monoenergetic synthesis are cubic interpolated from the NIST database^39^ at 1 keV intervals.

## Usage Notes

### Projection data

All projection data are stored as raw files with dimensions of 2063 × 505 pixels, in 16-bit unsigned integer format and little-endian byte order, where each pixel value represents the detected photon count. These files can be easily loaded and visualized using common software tools such as ImageJ^48^ and MATLAB. To facilitate data preprocessing, we provide several correction methods, including non-uniformity correction, ring artifact correction, and bad pixel interpolation. Users are also encouraged to implement and apply their own correction algorithms as needed. Dual-domain deep learning reconstruction techniques have demonstrated enhanced denoising capabilities in CT imaging^19^. This dataset enables users to train projection-domain Noise2Noise denoising models. For example, a single projection image can be used as the noisy input, while the average of its two adjacent projection images can serve as the self-supervised reference target.

### Reconstructed volumes

The reconstruction image size and resolution are fully customizable by users. In this work, we employed the TIGRE toolbox as the reconstruction framework. TIGRE offers a wide range of classical analytical and iterative reconstruction algorithms, including FDK, SART, MLEM, CGLS, FISTA, and total variation (TV) denoising. The analytical FDK algorithm is computationally efficient but typically results in higher image noise. To obtain high-quality images, users may opt for iterative reconstruction methods, which can be used as ground truth for training supervised deep learning models. Moreover, the dataset supports flexible low-dose reconstruction strategies. For example, images reconstructed from one sparse-angle subset can be used as inputs, while another subset or the full-dose reconstruction can serve as ground truth for Noise2Noise or Noise2Clear denoising model training.

### Spectral images

Here, we specifically decomposed the walnut shell and pulp as the two target materials. Due to the similarity in their attenuation coefficients, the decomposition matrix is ill-conditioned, resulting in significant noise amplification. To address this, users are advised to denoise the reconstructed high and low bin energy images prior to decomposition. We recommend using FDK followed by total variation (TV) denoising, or alternatively, iterative reconstruction methods to obtain high-quality CT images before decomposition. Image domain material decomposition is performed on a pixel-by-pixel basis. However, convolutional neural networks (CNNs) that leverage spatial information may further improve decomposition accuracy and reduce noise. Users may manually annotate high-quality base material maps of the shell and pulp to generate ground truth data for training decomposition models. Deep learning approaches can also be trained to directly predict virtual monoenergetic images at different energy levels.

In this dataset, we provide only the image-domain material decomposition method and its implementation. Theoretically, performing material decomposition in the projection domain could yield more accurate results, such as reducing beam-hardening effects. However, in our case, the primary goal is to separate the walnut shell and pulp. Projection-domain dual-material decomposition is challenging because the X-ray paths also pass through the PMMA holder, introducing a third basis material. Nevertheless, some studies have demonstrated projection-domain multi-material decomposition by incorporating additional constraints^49^, and we encourage users to further explore these approaches in this dataset. In addition, projection data acquired using different combinations of PMMA and aluminum slabs are provided, enabling users to perform projection-domain polynomial material decomposition for PMMA and AL^50,51^, and to synthesize virtual monoenergetic images. These data can also be used to calibrate the spectral response of the PCCT system^52^ and to develop model-based material decomposition algorithms.

### Further usage

This dataset supports a wide range of research applications, including detector uniformity correction, ring artifact removal, bad pixel interpolation, and validation of spectral reconstruction algorithms. It also serves as a flexible platform for simulating various types of artifacts to study broader challenges in spectral CT. Furthermore, users can perform pixel binning in the projection domain to explore super-resolution reconstruction methods.

Walnuts are commonly used in CT research due to their multi-scale structure, including a hard, symmetric shell, soft irregular kernel, and internal air cavities, which partially resemble features of animal head models^20,25^. This dataset provides high-resolution structural and spectral information valuable for developing and validating data-driven algorithms. Nevertheless, walnuts differ from animal and human tissues in density, composition, and X-ray attenuation, so algorithms trained solely on walnut data may require adaptation or calibration before application to preclinical or clinical datasets, and potential structural or spectral mismatches should be considered.

## Data Availability

The walnut photon-counting CT dataset generated and analyzed in this study is openly available on Zenodo (https://zenodo.org). The dataset is shared in partitioned bundles due to its total size (~296.8 GB), each assigned a unique DOI: calibration table&sample1 (10.5281/zenodo.15738313), sample2-3 (10.5281/zenodo.16163983), sample4-5 (10.5281/zenodo.16172256), sample6-7 (10.5281/zenodo.16176445), sample8-9 (10.5281/zenodo.16200172), sample10-11 (10.5281/zenodo.16200623), sample12-13 (10.5281/zenodo.16201067), and sample14-15 (10.5281/zenodo.16201185). The complete dataset includes: (1) CalibrationTable.zip, containing all calibration data required for preprocessing, reconstruction, and material decomposition; (2) CalibrationPhantomData.zip, including projection data of calibration phantoms (PMMA and aluminum slabs, water cylinder, and QRM HAP rods); (3) Reconstructions.zip, providing example reconstructed spectral and material-decomposed images of Walnut 1; (4) Walnut_1.zip – Walnut_15.zip, containing raw projection data and acquisition parameters for each walnut sample. All files are accessible via Zenodo’s web interface and can be downloaded individually.

## Code availability

All codes for dataset loading, correction, reconstruction, and material decomposition were implemented in MATLAB and have been made publicly available at: https://github.com/zezisme/WalnutPCCTReconCodes. Due to the large data volume, we recommend a machine with at least 64 GB of RAM and a GPU with ≥8 GB of memory. The repository provides two main modules: (1) projection data correction and reconstruction, and (2) image-domain spectral reconstruction. The reconstruction of low, high, and total energy bin images is performed using the WalnutDataRecon.m script, which relies on the open-source TIGRE toolbox (https://github.com/CERN/TIGRE). Users should download TIGRE main file and place it in a subdirectory of the “WalnutPCCTReconCodes” folder. Since TIGRE does not include precompiled MEX files by default, we provide precompiled mexw64 files in the “mexfiles” folder. Users may also recompile the MEX files based on their system configuration by following TIGRE’s instructions. The “WalnutDataRecon.m” script allows users to enable or disable non-uniformity and ring artifact corrections. It also supports the selection of reconstruction algorithms (e.g., FDK, iterative methods), filter types, angular sampling levels, and energy bins. For material decomposition tasks, where image noise significantly affects accuracy, we recommend applying FDK followed by 3D-TV denoising or using iterative reconstruction methods rather than standard FDK alone. Image-domain spectral reconstruction, including walnut material decomposition and virtual monoenergetic imaging (VMI), can be performed using the “WalnutSpectralRecon.m” script. Users can specify the desired monoenergetic energy levels via the “WalnutVMI_E” variable, which supports values ranging from 10 to 160 keV. All codes have been validated using MATLAB R2024a, and we recommend this version or newer for optimal compatibility.
